# Application of Artificial Neural Network and the Monomolecular Model in Describing the Relationship Between Body Weight Gain and Metabolizable Energy Intake in Egg‐Type Pullets

**DOI:** 10.1002/vms3.70887

**Published:** 2026-04-14

**Authors:** Zahra Moradi Gharajeh, Hassan Darmani Kuhi, Navid Ghavi Hossein‐Zadeh

**Affiliations:** ^1^ Department of Animal Science, Faculty of Agricultural Sciences University of Guilan Rasht Iran

**Keywords:** artificial neural network, body weight gain, egg‐type pullet, metabolizable energy, monomolecular model

## Abstract

**Background:**

Modelling growth allows nutritionists and poultry researchers to predict dynamic or daily nutrient needs more precisely than using fixed requirements.

**Objectives:**

This study evaluated the monomolecular model and artificial neural network (ANN) to describe the relationship between metabolizable energy (ME) intake and body weight gain (BWG) in commercial strains of different egg‐type pullets.

**Methods:**

A multi‐layer feed‐forward perceptron neural network structure was used to construct the ANN model. The best‐fitted network on input data to predict BWG from ME intake in all egg‐type pullets’ strains was obtained with one neuron in the input layer, three neurons in the first hidden layer, two neurons in the second hidden layer and one neuron in the output layer, which was written as 1‐3‐2‐1.

**Results:**

The relationship between ME intake and BWG in different egg‐type pullet strains was predicted with very high accuracy by both ANN (*R* ^2^ adj = 99.47–99.99) and the monomolecular model (*R* ^2^ adj = 98.48–99.72). The maintenance energy requirements (134.2–165.8 kcal/kg BW) and efficiencies of NE utilization for growth (2.23–4.00 kcal/g of BW) estimated by the monomolecular model are consistent with previously reported values for poultry. Meta‐analysis of the parameters estimated by the monomolecular model (a, b and c) revealed significant differences among strains (*p* < 0.01), suggesting that these strain‐specific growth responses may be linked to genetic diversity.

**Conclusions:**

This study demonstrated that both the monomolecular model and ANN approaches effectively described the relationship between ME intake and BWG in egg‐type pullets.

## Graphical abstract

Energy utilization in egg‐type pullets is shaped by genetic diversity and body‐weight dynamics. A simple mechanistic model captured interpretable energy–growth relationships, while artificial neural networks improved predictive accuracy, highlighting complementary modelling approaches for understanding and predicting pullet energy dynamics.

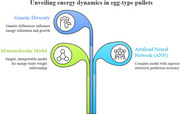

## 1 Introduction

Dietary energy is one of the nutritional factors that greatly affects the growth rate of animals. Therefore, paying attention to the ME level of the diet will have a positive effect on the economic production of poultry (Dozier and Moran 2001).

On the other hand, experiments designed to investigate the effect of nutrient concentration in the diet on the growth and development of pullets are relatively long‐term and costly. As research costs increase, mathematical models become valuable tools for answering research questions. Therefore, adequate knowledge and forecasting of nutrient requirements have many economic benefits.

Non‐linear models were used to partition ME intake to maintenance and growth requirements in ruminants, broilers and turkeys. These models are important because of their ability to predict ME dietary requirements for daily maintenance and growth throughout the growing period. The parameters derived from these growth models provide biologically meaningful information for describing complex biological relationships. These biologically interpretable parameters can also be used to compare energy utilization efficiency and growth patterns among different populations or strains (Narinç et al. 2017).

In addition to non‐linear growth models such as the monomolecular model, artificial neural networks (ANNs) have been used to predict growth patterns and growth rates in living organisms and have attracted considerable attention in computational methods (Roush et al. 2006). ANNs are powerful tools that can describe complex relationships between inputs and outputs in a database without prior knowledge of functional relationships (Dayhoff and DeLeo 2001). This characteristic makes ANN particularly suitable for modelling nonlinear interactions between dietary energy intake and body weight, where traditional parametric models may be limited. Similarly, recent work in quail chicks has applied ANN to predict growth responses to dietary energy levels, reinforcing the utility of neural networks in nutritional modelling (Ghazaghi et al. 2025).

The inherent nature of neural networks is that they process information in parallel and simultaneously, and this has increased the speed of calculations and their use in solving different problems simultaneously (Bishop and Nasrabadi 2006). Multi‐layer neural networks act as powerful nonlinear models with the ability to learn complex relationships from input to output variables by combining non‐linear activation functions and communication of different weights in different layers. These networks can learn and recognize more complex patterns and features from data. This ability allows them to improve a variety of issues, from pattern recognition to forecasting and decision‐making.

The powerful potential and flexibility of ANN models for solving complex nonlinear problems, control problems and predicting important economic traits such as egg production (Faridi et al. 2013), growth and reproductive performance (Mehri 2013) and nutritional requirements (Ahmadi and Golian 2010; Mehri 2014) have been reported previously.

In addition to predictive modelling approaches, accurate estimation of model parameters is essential for improving biological interpretation and decision‐making. Accurate estimation of genetic parameters is essential for setting selection plans in breeding programs. Over the past years, various studies reported the genetic parameters for different growth, reproduction and egg quality traits in Iranian native fowls. These assessments were obtained using a variety of methods and from studies of different populations of native fowls, leading to significant variation in genetic parameter estimates.

This study aimed to perform a meta‐analysis based on a random‐effects model to overcome the diversity of reported genetic parameters for economically important traits of Iranian native fowls (Gholipour et al. 2023).

The growth curve is widely used to design feeding programs suitable for age and to determine selection in breeding programs (Abbas et al. 2014; Al‐Samarai 2015).

The growth rate of sheep in terms of the shape of the growth curve can be explained using mathematical equations, and further, the important biological parameters of the growth curve can be interpreted (Da Silva et al. 2012). These parameters describe the relationship between body weight and age, which can be used to obtain an optimal growth curve in a desired population (Bathaie and Leroy 1998; Malhado et al. 2009).

Previous authors have studied the characteristics of the growth curve using different non‐linear growth models (Gbangboche et al. 2008; Kopuzlu et al. 2014; Hossein‐zadeh 2015a; Mokhtari et al. 2019) and showed that knowledge of growth curve parameters may be used to maintain a healthier herd structure, decide on slaughter age, cull poor animals and optimize feeding strategies for greater on‐farm profit.

Furthermore, genetic evaluation of growth curve parameters can be performed to distinguish genetic variation among animals from total variation to determine more precise selection criteria (Bathaie and Leroy 1998). This may have greater potential for assessing response to selection over time, identifying superior germplasm in advance, and optimizing breeding strategies (Lambe et al. 2006; Hojjati and Hossein‐Zadeh 2018). Therefore, accurate estimation and synthesis of growth curve parameters are essential to ensure reliable genetic interpretation and effective selection decisions.

Meta‐analysis is a method of synthesis of quantitative data from multiple independent studies addressing a common research question. An important part of this method involves computing a combined effect size across all of the studies. As such, this statistical approach involves extracting effect sizes and variance measures from various studies (Kelley and Preacher 2012).

By combining these effect sizes, the statistical power is improved and can resolve uncertainties or discrepancies found in individual studies. Meta‐analysis can support or lead to the rejection of a proposed hypothesis by gathering and scrutinizing data sources. Ideally, meta‐analysis permits the aggregation of lesser‐powered studies into one analysis to detect a significant difference between treatments. In other words, this is a research technique that tries to combine different studies on the same topic to provide a quantitative result that might address the questions under study. Accordingly, meta‐analysis provides an appropriate framework for integrating growth model parameter estimates across multiple strains.

The present study aimed to evaluate and compare the ANN and monomolecular model predictive ability for forecasting the ME requirements from BWG in egg‐type pullets' strains. In addition, a meta‐analysis was conducted on parameters estimated by the monomolecular model, as these parameters have biological relevance, to investigate the possibility of significant differences that may exist among different strains and may be correlated with genetic diversity.

Despite previous studies on growth parameters and predictive modelling in poultry, there is still a lack of integrated information on the relationship between metabolizable energy (ME) intake and body weight gain (BWG) across multiple strains of egg‐type pullets. This study combines nonlinear growth modelling and ANNs to predict the relationship between ME intake and BWG in different strains. Furthermore, a meta‐analysis of growth model parameters of the monomolecular was performed across six different egg type strains of pullets, which has not been addressed in previous studies.

## 2 Materials and Methods

In this study, data derived from six commercial laying hen strain management databases (Hy‐Line W36, ISA Brown, Dekalb White, Bovans White, Bovans Brown, and Shaver; HGC 2025a‐e, Hy‐line 2020) were used to assess the predictive performance of a monomolecular function (Equation 1) and an ANN in describing the relationship between ME intake and BWG. The datasets corresponded to laying hen pullets during the rearing period from 1 to 18 weeks of age. All records were extracted from published management guides and commercial documentation, entered into Excel spreadsheets and checked for accuracy and internal consistency before analysis. Model fitting was performed using the nonlinear regression procedure of SigmaPlot for traditional functions and the ANN module of GMP_pro_ software for neural network analysis. This research was conducted exclusively using previously published and commercial data sources, and no new animal experimentation was involved. Consequently, additional institutional animal care or ethics approval was not required for this secondary data analysis.

(1) $$Y=a-\left(a\;+b\right)e^{-cx}$$

where the parameters *a*, *b* and *c* are positive entities, with *y*_max_ = *a* and *y*_min_ = −*b*.

BWG (g/g) and ME intake (kcal/kg) were calculated for each data profile as follows:

(2) $$\mathrm{BW}\;\mathrm{gain}\;=\frac{\mathrm{Difference}\;\mathrm{of}\;\mathrm{final}\;\mathrm{and}\;\mathrm{initial}\;\mathrm{pullet}\;\mathrm{BW}\;\mathrm{each}\;\mathrm{wee}k}{\left(\overset{⇀}{\mathrm{BW}}\right)\times \;\mathrm{days}\;\mathrm{of}\;\mathrm{rearing}}$$

(3) $$\mathrm{ME}\;\mathrm{intake}\;=\frac{\mathrm{ME}\;\mathrm{intake}\;\mathrm{for}\;a\;\mathrm{specific}\;\mathrm{week}}{\left(\overset{⇀}{\mathrm{BW}}\right)\times \;\mathrm{days}\;\mathrm{of}\;\mathrm{rearing}}$$

where $\bar{\mathrm{BW}\;}$ is average BW. Average BW for different ages was calculated as the mean of the weight at the hatch and the end of each week. If the initial (hatch) BW was not recorded, a value of 38 g was assumed.

In this study, a multilayer perceptron (MLP) feed‐forward ANN model was constructed using the ANN module of JMP Pro software to predict the relationship between ME intake and BWG in pullets. The used MLP ANN consists of 4 layers: One input layer, two hidden layers and one output layer. The activation function used in the hidden and output layers was a hyperbolic tangent activation function using a fixed 33% validation data set. The output of the model (BWG) was predicted, and the resulting prediction was compared with their observed values. Different statistical criteria, including adjusted coefficient of determination (*R* ^2^), root mean square error (RMSE), mean absolute deviation (MAD), mean absolute percentage error (MAPE), Akaike's information criterion (AIC) and Bayesian information criterion (BIC), were used to evaluate the suitability of the monomolecular equation and the ANN model. The statistical criteria were calculated as follows:

(4) $$R^{2}\text{adj}=1-\left[\left(n-1/n-p\right)\left(1-R^{2}\text{model}\right)\right]$$

where *R* ^2^ adj is the adjusted coefficient of determination, *n* is the number of observations, *p* is the number of parameters in the model and *R* ^2^ is the coefficient of determination of the model. This coefficient is the RSS/TSS ratio, where RSS is the residual sum of squares and TSS is the total sum of squares.

(5) $$\text{RMSE}=\sqrt{(}\text{RSS}/n-p)$$

where RSS is the residual sum of squares, *n* is the number of observations and *p* is the number of parameters in the model.

(6) $$\text{MAD}=\sum (A_{i}-F_{i})/n$$

(7) $$\text{MAPE}=1/n\sum |\left(A_{i}-F_{i}\right)/A_{i}|\times 100$$

(8) AIC=n×ln(RSS)+2P

(9) BIC=nln(RSS/n)+pln(n)

where *A_i_* is the actual value, *F_i_* is the forecasted value, RSS is the residual sum of squares, *n* is the number of observations and *p* is the number of parameters in the models. Except *R* ^2^ adj where higher values indicate a better fit of the model to the data, for the remaining studied criteria, the best model is the one with the lowest values.

A meta‐analysis of the estimated parameters of the monomolecular model was conducted across the different strains to examine potential differences between them. These findings could contribute to the development of strategies for optimizing production processes and genetic improvement efforts. For meta‐analysis, we used “CMA” software (Biostat, Englewood, NJ, USA V.3) based on the random‐effects model. Heterogeneity between the parameter estimates was assessed using the *I* ^2^ test and Cochran's *Q*. A high *I* ^2^ value indicates the presence of heterogeneity. In general, values ​​of 25%, 50% and 75% are interpreted as low, moderate and high heterogeneity, respectively.

(10) $$I^{2}=\left(Q-\left(K-1\right)/Q\right)\times 100$$

In this equation, *Q* was the Cochran heterogeneity statistic, and *K* was the number of trials.

## 3 Results and Discussion

**FIGURE 1:**
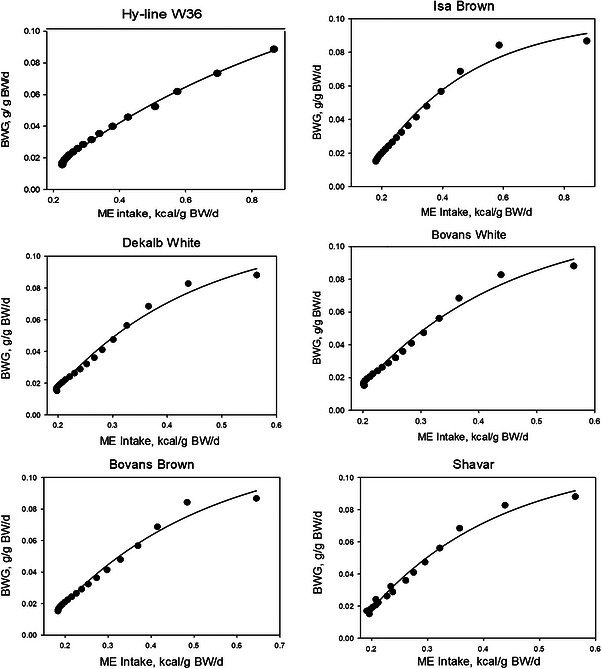
Plots of ME intake (kcal/g of BW per day) against BW gain (g/g of BW per day), showing fits of the monomolecular model to data from different varieties of egg‐type pullets.

The results of fitting the data with the monomolecular model (Figure 1) show that the model adequately describes the relationship between ME intake and BW gain in egg‐type pullets. Tables 1 and 2 present the statistical performance of the model and the biological interpretability of the estimated parameters. Based on the reported statistical criteria, the overall fit of the model to the data was acceptable. According to the parameter estimates presented in Table 2, the ME requirement for maintenance ranged from 134.2 to 165.8 kcal/kg BW across different strains. In addition, the estimated average use of NE for body growth between one‐ and four‐times maintenance ranged from 2.23 to 4.00 kcal/g of BW among strains. These differences likely reflect genetic variation in maintenance requirements and growth efficiency among different egg‐type pullet strains. The estimated maintenance energy requirements and efficiencies of NE utilization for growth are consistent with previously reported values for poultry, supporting the biological validity of the parameter estimates obtained from the monomolecular model (Darmani Kuhi et al. 2004; Sakomura 2004).

**TABLE 1:** Parameter estimates obtained with a monomolecular model to regress BW gain (g/g BW) to ME intake (kcal/g BW).

Parameter estimate				
Variety	*a*	*b*	*c*	*R* ^2^ ^a^
Hy‐Line W36	0.0861(0.0030)	0.0219 (0.0033)	2.2129 (0.2001)	99.72
Isa Brown	0.1006(0.0052)	0.1342(0.0028)	3.1921(0.3771)	98.48
Dekalb White	0.1162(0.0096)	0.1615(0.0048)	3.9070 (0.6134)	98.55
Bovans White	0.1166(0.0100)	0.1658(0.0049	3.9254(0.6343)	98.48
Bovans Brown	0.1201(0.0131)	0.1351(0.0076)	2.8148(0.5592)	97.57
Shavar	0.1116 (0.0084)	0.1593(0.0047)	4.2621 (0.6419	98.49

*Note*: Standard errors are given in parentheses.

^a^ Adjusted *R* ^2^.

**TABLE 2:** Growth traits calculated from parameter estimates obtained with a monomolecular model to regress BW gain (g/g BW) to ME intake [kcal/g BW].

Variety	MEm (kcal/kg BW)	NEg^a^ (kcal/g BW)	kg (1)^b^	kg (2)^b^	kg (3)^b^	kg (4)^b^
Hy‐Line W36	135.6	4.00	0.21	0.19	0.16	0.15
Isa Brown	134.2	2.92	0.32	0.27	0.23	0.20
Dekalb White	161.5	2.29	0.45	0.35	0.29	0.26
Bovans White	165.8	2.29	0.46	0.35	0.29	0.26
Bovans Brown	135.1	2.68	0.34	0.28	0.25	0.22
Shavar	159.3	2.23	0.48	0.36	0.30	0.27

^a^ NEg = The average net energy requirement for growth between 1 and 4 times maintenance, calculated as 0.62 / *kg* (1–4) based on the assumption that the average efficiency of utilization of ME for growth is approximately 0.62 % for balanced diets in egg‐type pullets (Sakomura 2004).

^b^ The average efficiency of utilization of ME for growth [g of BW gain/kcal of ME intake] between 1 and 2 and 3 and 4 times maintenance.

ANN models offer an alternative approach to traditional regression methods for investigating complex biological systems. A key advantage of ANN models is that they do not require an a priori equation to define the relationship between input and output variables, which is particularly useful when nonlinear responses are expected (Roush et al. 1997).

**FIGURE 2:**
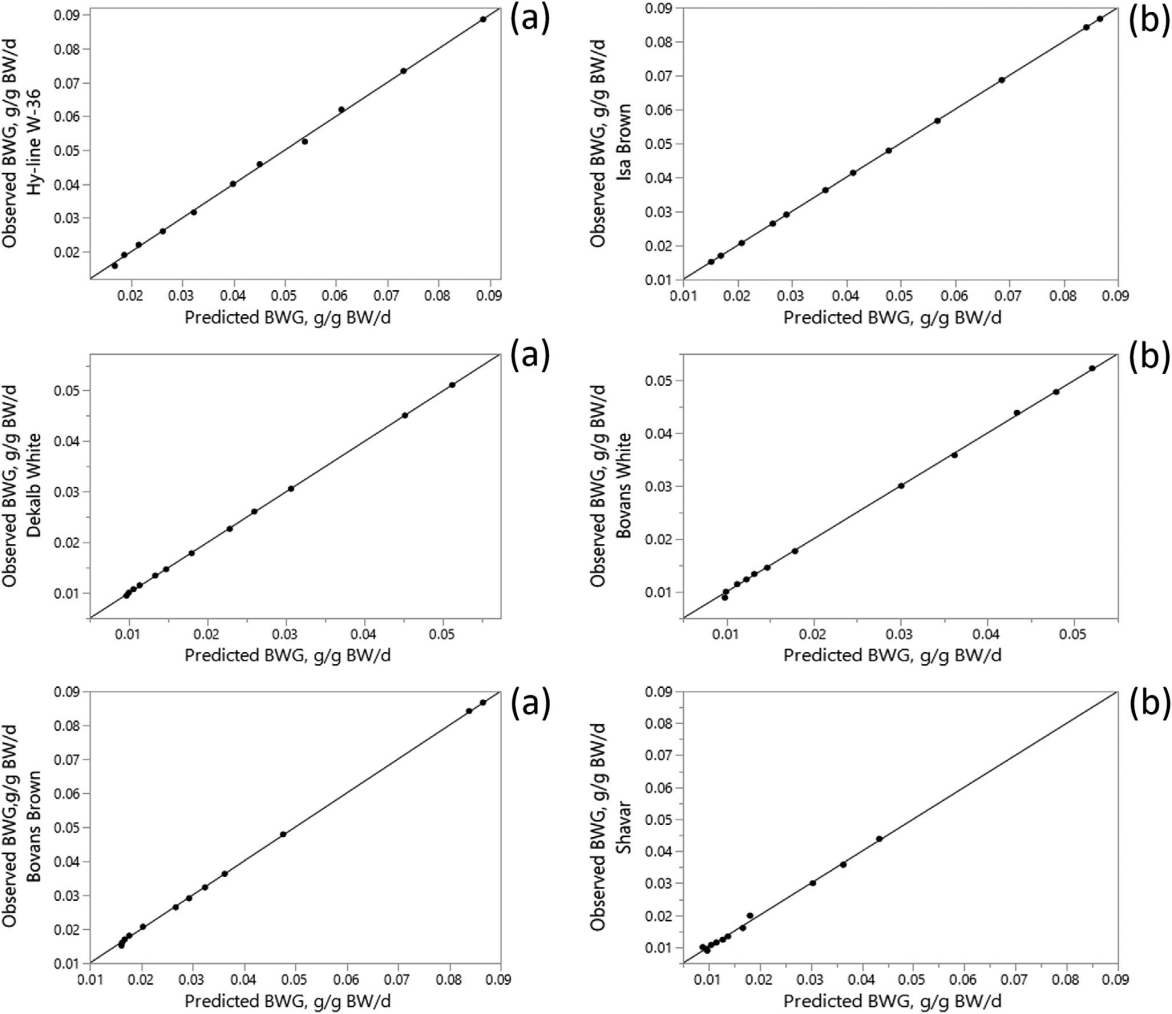
Observed versus predicted plot by artificial neural network for training data sets.

**FIGURE 3:**
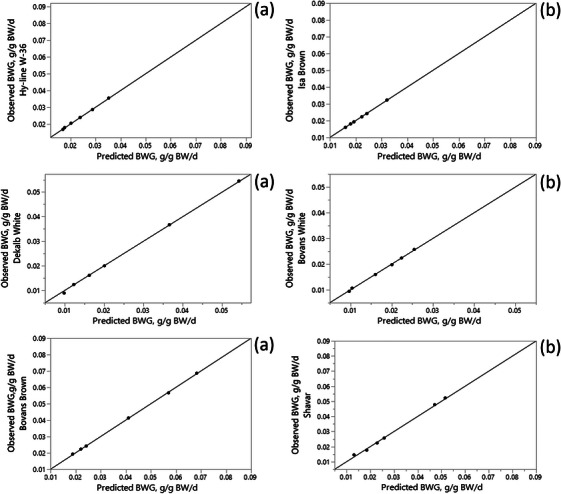
Observed versus predicted plot by artificial neural network for validating data sets.

The predictive performance of the ANN model in describing the relationship between ME intake and BWG was evaluated using *R* ^2^, RMSE, MAD and MAPE criteria, as presented in Table 3. Figures 2 and 3 illustrate the relationship between observed and predicted BWG values across different levels of ME intake. The agreement between predicted and observed values indicates that the ANN model was able to effectively capture the input–output relationship. This close correspondence between predicted and observed BWG values demonstrates the ability of the ANN model to represent the input–output relationship. ANNs have been widely applied to model and predict the behaviour of complex or partially unknown systems based on input–output data. This feature makes ANN models particularly advantageous for modelling biological systems (Dayhoff and DeLeo 2001). In many cases, ANN predictions result in a closer fit to experimental data than conventional regression approaches. Consistent with this expectation, the results of the present study showed that for most statistical criteria, except AIC and BIC, the ANN model outperformed the monomolecular model in describing the response of pullets to ME intake for both the training and validation data sets (Table 3). However, based on the AIC and BIC criteria, the monomolecular model showed superior performance compared with the ANN model (Table 3). This difference can be attributed to the lower number of parameters and reduced model complexity of the monomolecular model. Similar results were reported by Ahmadi and Golian (2011), who found higher prediction accuracy of ANN models for weight gain and feed conversion ratio in broiler chickens compared with response surface methodology. Mehri et al. (2012) demonstrated that MLP ANN models could predict broiler performance and estimate digestible amino acid requirements with high accuracy. In addition, Jahan et al. (2020) reported that ANN models provided a practical approach for predicting the final body weight of Japanese quails based on early growth performance.

**TABLE 3:** Statistics criteria of the Monomolecular model and artificial neural network (ANN) for predicting BW gain from ME intake.

		Hy‐LINE W36	Isa Brown	Dekalb White	Bovans White	Bovans Brown	Shavar
	—	**Monomolecular model**					
MAD^a^	—	0.00005	0.00011	0.00012	0.00011	0.00011	0.00011
RMSE^b^	—	0.000005	0.000026	0.000026	0.000026	0.000026	0.000026
MAPE^c^	—	3.369	5.286	5.655	5.557	4.794	5.655
AIC	—	−177.59	−159.78	−150.57	−150.57	−150.57	−150.57
BIC	—	−223.26	−196.24	−196.24	−196.24	−197.04	−196.24
	—	**Artificial neural network**					
*R* ^2^	Training set	0.99866	0.99988	0.99994	0.99991	0.99969	0.9947
	Validating set	0.99792	0.99967	0.99996	0.99650	0.99982	0.9955
MAD^a^	Training set	0.00064	0.00020	0.00030	0.00016	0.00030	0.00128
	Validating set	0.00053	0.00009	0.00040	0.00051	0.00026	0.00079
RMSE^b^	Training set	0.00087	0.00026	0.00049	0.00023	0.00037	0.00177
	Validating set	0.00062	0.00010	0.00047	0.00081	0.00032	0.00098
MAPE^c^		2.23	0.67	1.59	1.43	1.25	4.06
						
AIC^d^	Training set	−108.60	−148.31	−132.80	−151.25	−139.80	−102.23
	Validating set	−57.87	−78.98	−61.00	−54.66	−65.57	−52.32
BIC^e^	Training set	−130.99	−173.28	−157.77	−176.22	−164.70	−127.20
	Validating set	−70.70	−91.82	−73.83	−67.49	−78.40	−65.15

^a^ Mean absolute deviation.

^b^ Root mean squared error.

^c^ Mean absolute percentage error.

^d^ Akaike's information criterion.

^e^ Bayesian information criterion.

**FIGURE 4:**
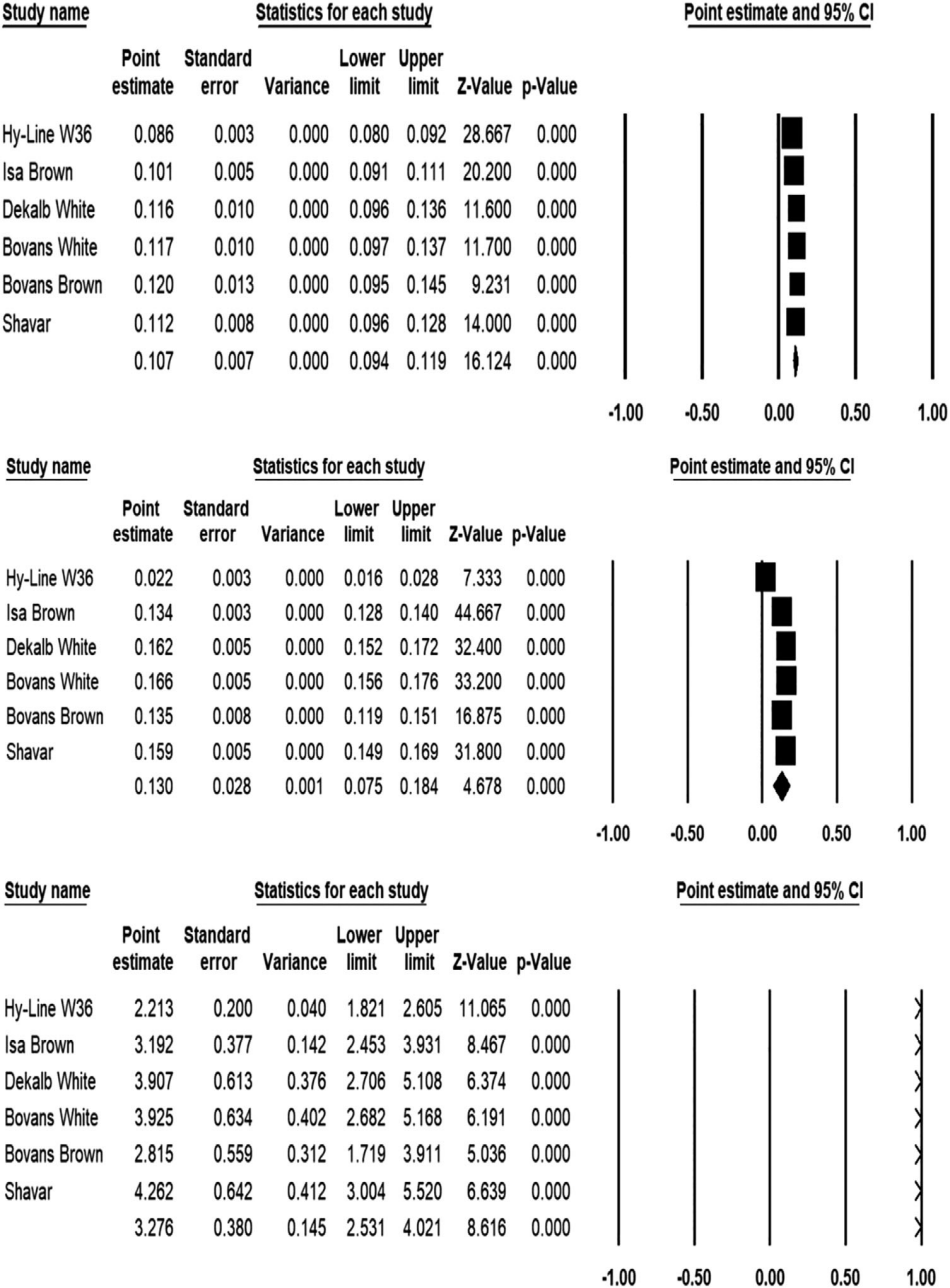
The forest plot presents the results of the meta‐analysis for parameters of the monomolecular model (a, b and c). Each study is represented by a square, with its size proportional to the study's weight in the overall analysis. The horizontal bars extending from each square represent the 95% confidence intervals (CIs) of the estimates, providing a measure of the precision of each study's effect size. The mean effect size, calculated according to a random‐effects model, is indicated by the diamond at the bottom of each plot. The plot clearly illustrates both the individual study effects and the overall summary estimate, highlighting the statistical significance and heterogeneity across studies.

A summary of the meta‐analysis results comparing parameters estimated by the monomolecular model across different strains is presented in Figure 4. Figure 4 presents forest plots of the monomolecular model parameters (*a*, *b* and *c*), showing the corresponding confidence intervals, standard errors and the overall pooled estimates from the meta‐analysis. The results of the heterogeneity analysis for each parameter are summarized in Table 4. The *I* ^2^ values for parameters *a*, *b* and *c* were estimated to be 82.05%, 99.60% and 76.34%, respectively. Because all *I* ^2^ values exceeded 50%, a random‐effects model was selected for the meta‐analysis. The random‐effects model accounts for both within‐study and between‐study sources of variability in the pooled estimates (Vesterinen et al. 2014). The high *I* ^2^ values indicate substantial heterogeneity among studies for all three parameters, which is likely attributable to genetic differences among the evaluated strains.

**TABLE 4:** Result of meta‐analyses and heterogeneity test for the studied parameters.

	Effect size and 95% confidence interval					Heterogeneity analysis		
Parameters	Point estimate	Standard error	Lower limit	Upper limit	*p*‐value	*Q*‐value^a^	*p*‐value^b^	*I*‐squared^c^
*a*								
Random effects models	0.107	0.007	0.094	0.119	0.000	27.868	0.000	82.058
*b*								
Random effects models	0.130	0.028	0.075	0.184	0.000	1263.308	0.000	99.604
*c*								
Random effects models	3.276	0.380	2.531	4.021	0.000	21.137	0.001	76.344

a The Cochran test is a test of heterogeneity (a high value of *Q* and a low *p*‐value indicates heterogeneity of studies).

b *p*‐value relates to the Cochran (*Q*) test.

c *I* ^2^ statistics, heterogeneity test (if the values of *I* ^2^ are equal to 25%, 50% and 75%, it is interpreted as low, medium, and high heterogeneity, respectively).

## 4 Conclusion

This study demonstrated that both the monomolecular model and ANN approaches are effective in describing the relationship between ME intake and BWG in egg‐type pullets. Although both models provided acceptable predictions, the ANN showed superiority to the monomolecular model based on most statistical criteria, but the monomolecular model offered greater simplicity and biological interpretability of parameters. The meta‐analysis on the estimated monomolecular model revealed considerable differences among different egg type strains of pullets, highlighting the role of genetic diversity in energy utilization and growth performance, which supports the need to use strain‐specific nutritional strategies.

## Author Contributions

**Zahra Moradi Gharajeh**: methodology, formal analysis, data curation. **Hassan Darmani Kuhi**: writing – original draft, methodology, formal analysis, conceptualization. **Navid Ghavi Hossein‐Zadeh**: writing, editing, formal analysis.

## Funding

The authors have nothing to report.

## Ethics Statement

The authors have nothing to report.

## Consent

The authors have nothing to report.

## Data Availability

The data that support the findings of this study are available from the corresponding author upon reasonable request.
